# Global Burden of Dental Caries and Oral Disorders: A 31-Year Comparative Analysis of Trends in Iran, North Africa and Middle East

**DOI:** 10.34172/aim.33209

**Published:** 2025-06-01

**Authors:** Saeed Asgary, Alireza Akbarzadeh Baghban, Fatemeh Mahmoudi Afsah

**Affiliations:** ^1^Iranian Center for Endodontic Research, Research Institute of Dental Sciences, Shahid Beheshti University of Medical Sciences, Tehran, Iran; ^2^Proteomics Research Center, Department of Biostatistics, School of Allied Medical Sciences, Shahid Beheshti University of Medical Sciences, Tehran, Iran

**Keywords:** Dental caries, Dental public health, Disability-adjusted life years, Epidemiology, Global health, Incidence, Oral disorders, Prevalence

## Abstract

**Background:**

The Global Burden of Disease (GBD) studies highlight oral health as a significant global concern, particularly in regions such as Iran, the Middle East and North Africa (MENA). This study synthesizes GBD data on the incidence, prevalence, and disability-adjusted life years (DALYs) associated with dental caries and oral disorders to inform targeted interventions aimed at alleviating their burden and improving outcomes in these regions.

**Methods:**

This study employs estimation techniques to comprehensively assess the occurrence and impact of dental caries in deciduous and permanent teeth, along with oral disorders across Iran, MENA, and globally. Data were drawn from the GBD site. We analyzed incidence, prevalence, and DALYs, utilizing disability weights with joinpoint regression models to inform public health interventions.

**Results:**

From 1990 to 2021, global and regional trends in dental caries and oral disorders were examined. Descriptive statistics illustrate significant regional disparities in incidence, prevalence, and DALYs. Globally, the incidence rate of caries in deciduous teeth decreased at an average annual percent change (AAPC) of -0.25% (95% CI: -0.30 to -0.20), while the MENA region and Iran showed AAPCs of -0.57% (-0.65 to -0.49) and -0.08% (-0.15 to -0.01), respectively. These trends highlight both progress and persistent disparities. The models reveal temporal changes in disease burden, with notable declines and fluctuations over time. These findings underscore persistent challenges in oral health, particularly in regions such as Iran and MENA, where socioeconomic conditions and healthcare access vary significantly.

**Conclusion:**

This study underscores considerable regional variation in dental caries and oral disorders, emphasizing ongoing public health challenges. Demographic shifts and reduced sugar intake, influenced by sociopolitical factors like war, have significantly decreased the global burden of oral conditions since 1990. The findings underscore the urgency of integrating oral health into primary care systems, expanding subsidized fluoride programs in rural areas, and addressing socioeconomic barriers to dental access in MENA. Community-driven initiatives, such as school-based screenings and mobile dental clinics, should be prioritized in underserved regions.

## Introduction

 Oral health is essential to overall well-being and quality of life, yet it remains a significant global public health issue. Dental caries and other oral disorders are widespread, affecting millions and imposing substantial morbidity and economic burdens. Despite advancements in dental care, these conditions continue to negatively impact quality of life, productivity, and health outcomes, particularly in regions with limited access to dental services.^1-4^

 The high burden of oral disorders in the Middle East and North Africa (MENA) directly impedes progress toward SDG 3 (Good Health and Well-being), particularly Target 3.8 (Universal Health Coverage). Untreated dental conditions exacerbate poverty cycles by limiting educational attainment and workforce participation, further entrenching health inequities.

 Global Burden of Disease (GBD) studies offer a comprehensive framework for understanding the epidemiology and burden of oral health conditions worldwide. By analyzing data on incidence, prevalence, and disability-adjusted life years (DALYs), these studies provide critical insights into the distribution, trends, and disparities in oral health outcomes across different regions and demographic groups.

 Dental caries, one of the most prevalent and preventable oral health issues, can range from minor subclinical changes to severe tooth destruction. Untreated dental caries can lead to considerable pain, infection, and functional impairment, especially among children and adolescents.^5-8^ Similarly, other oral disorders, including periodontal disease and oral cancer, significantly impact overall health and well-being.^6,8-10^

 Despite the comprehensive data provided by GBD studies, disparities in oral health persist, particularly in regions like Iran and MENA. These areas often face challenges related to socioeconomic conditions, healthcare infrastructure, and access to preventive and therapeutic dental services. Addressing these regional disparities is critical for developing effective public health strategies and appropriately allocating resources.

 In MENA, oral diseases disproportionately affect marginalized populations, aligning with the emphasis of SDG 3 on reducing inequalities. For example, the prevalence of childhood caries in low-income MENA communities is 3–4 times higher than urban centers, reflecting systemic gaps in healthcare access. For instance, in conflict-affected Yemen, only 12% of rural populations have access to dental care, compared to 45% in urban areas. Economic sanctions in Iran have reduced imports of dental materials by 30%, worsening treatment delays.

 We hypothesize that socioeconomic inequities, fragmented healthcare systems, and insufficient preventive policies drive higher oral disease burdens in MENA compared to global averages. This study evaluates these factors through trend analysis and proposes region-specific interventions.

 This study synthesizes GBD data on the incidence, prevalence, and DALYs associated with dental caries in deciduous and permanent teeth, as well as other oral disorders, both globally and specifically in Iran and MENA. By examining epidemiological trends over a 31-year period, this study aims to elucidate the current state of oral health in these regions, identify key challenges, and propose opportunities for intervention.

## Materials and Methods

 This observational study was approved by Ethics Committees of Research Institute of Dental Sciences, Shahid Beheshti University of Medical Sciences. The study utilized data obtained from the GBD website. Consequently, an ethical statement for research involving human or animal subjects is not applicable.

###  Obtaining and Estimation Process of GBD 

 Given the variability in data across different geographical areas, the GBD study employs various estimation techniques to ensure thorough coverage (https://www.healthdata.org/research-analysis/gbd).^11^ Data sources encompass dental health surveys, health facility records, and oral health databases spanning regions such as Iran, MENA, and Global surveys.^1-3,12-14^ The estimation process begins with collecting data on caries incidence, prevalence in both deciduous and permanent teeth, and oral disorders. Where DALY data are insufficient, models project the burden by integrating incidence and severity. These projections are vital for understanding the public health impact of caries in deciduous teeth.

 DALYs are employed to evaluate the impact of caries, combining years of life lost (YLL) due to premature mortality and years lived with disability (YLD) from non-fatal health outcomes.^2,15^ Incidence and prevalence data inform DALY estimates, alongside disability weights, reflecting the severity of caries varying according to disease progression and treatment stages.^1,2,15^ This methodological approach facilitates a comprehensive evaluation of caries’ public health impact, guiding effective resource allocation and intervention strategies.

###  Search Strategy and Data Extraction

 Data on the prevalence of dental caries and oral disorders in Iran, MENA, and globally were obtained from the GBD Study 2021. The study employed metrics like incidence, prevalence, and DALYs, integrating YLL and YLD, to assess these conditions. This comprehensive analysis informs healthcare policy development and resource allocation across these regions.

###  Statistical Analysis

 According to the requested indicators, the result files from GBD 2021 were received and saved in Excel sheets. While GBD models standardize data across regions, MENA-specific limitations include underreporting in conflict zones (e.g. Syria, Libya) and variability in national survey methodologies. These factors may introduce uncertainty, particularly in rural and displaced populations.

 GEE was used to compare locations. The present study utilized Joinpoint regression analysis with Joinpoint software (Joinpoint Version 4.8.0.1) to investigate temporal trends in incidence, prevalence, and DALYs, employing annual percent changes (APCs) and average annual percent changes (AAPC). Joinpoint regression is a statistical method employed to detect and analyze shifts in trends over time by identifying the joinpoint where significant changes in trend slopes occur. While joinpoint regression effectively identifies trend shifts, it assumes linearity between joinpoints and may oversimplify complex temporal patterns. Additionally, GBD estimates rely on modeled data, which may underrepresent subnational disparities in MENA due to sparse primary data. Joinpoint regression was selected for its ability to objectively identify trend inflection points. However, it may not capture gradual, non-linear changes. We mitigated this by using bootstrapping to validate joinpoint locations. This approach assumes that the observed data can be represented as a series of connected line segments, each characterized by a distinct slope. The number and locations of joinpoint are estimated based on statistical criteria. The joinpoint regression process involves the following procedural steps:

1. Data should be sequentially ordered, with the dependent variable measured across time intervals.
2. Apply an algorithm to fit regression models, identifying optimal joinpoints that minimize residual sum of squares.

Estimate joinpoint locations and slope changes, assessing their statistical significance through hypothesis testing.

## Results

Table 1 outlines the descriptive statistics for incidence, prevalence, and DALYs related to caries in deciduous and permanent teeth, as well as oral disorders, from 1990 to 2021. This table offers a comprehensive overview of epidemiological trends, highlighting the global burden and regional variations over three decades.

**Table 1:** The Incidence, Prevalence and DALY Rate per 100 000 Along with Uncertainty Intervals of Deciduous Teeth Caries, Permanent Teeth Caries and Oral Disorders in Three Locations for the Selected Years from the Global Burden of Disease Database

			**1990**	**1995**	**2000**	**2005**	**2010**	**2015**	**2021**
Caries of deciduous Teeth	Incidence	Global	19290.73 (14361.4, 26873.0)	19056.57 (14311.3, 26756.2)	18950.66 (14226.7, 26158.6)	18430.59 (13927.0, 25135.5)	18026.90 (13694.5, 24262.5)	18038.78 (13757.4, 24004.3)	17781.15 (13952.3, 23035.4)
		MENA	19388.28 (14296.4, 27471.3)	19200.82 (14204.0, 27178.9)	18844.05 (14261.2, 26237.4)	17438.07 (13788.0, 22174.6)	15963.01 (12672.4, 19887.8)	16104.75 (12606.1, 19986.7)	16170.92 (12510.7, 20082.2)
		Iran	20813.73 (15184.1, 29450.6)	20435.33 (14747.0, 29192.8)	20429.45 (14838.1, 29376.8)	20416.58 (15067.6, 29068.9)	20356.56 (14954.1, 28758.2)	20302.81 (15113.2, 28671.3)	20255.81 (15081.0, 28789.8)
	Prevalence	Global	8105.37 (6573.4, 9809.0)	8009.54 (6509.9, 9687.1)	7929.92 (6482.0, 9532.5)	7776.15 (6402.3, 9298.2)	7620.13 (6329.8, 9058.6)	7588.34 (6313.9, 8975.5)	7548.04 (6290.6, 8775.8)
		MENA	7921.17 (6330.4, 9587.1)	7931.12 (6318.3, 9695.2)	7886.79 (6337.4, 9538.3)	7792.38 (6592.5, 9140.3)	7517.92 (6497.2, 8565.0)	7502.77 (6476.3, 8582.6)	7446.43 (6242.5, 8668.0)
		Iran	8866.92 (7340.2, 10551.1)	8829.99 (7177.3, 10656.6)	8818.04 (7108.4, 10666.0)	8825.27 (7214.8, 10545.2)	8766.39 (7222.5, 10453.3)	8838.16 (7283.2, 10511.5)	8806.36 (7381.7, 10390.8)
	DALY	Global	3.09 (1.3, 6.1)	3.06 (1.3, 6.0)	3.03 (1.3, 5.9)	2.97 (1.2, 5.7)	2.91 (1.2, 5.6)	2.90 (1.2, 5.5)	2.89 (1.2, 5.5)
		MENA	3.03 (1.2, 6.0)	3.04 (1.3, 5.9)	3.02 (1.2, 5.8)	2.99 (1.3, 5.6)	2.88 (1.3, 5.6)	2.88 (1.3, 5.6)	2.85 (1.3, 5.5)
		Iran	3.39 (1.4, 6.6)	3.38 (1.4, 6.6)	3.38 (1.4, 6.4)	3.38 (1.4, 6.4)	3.36 (1.4, 6.4)	3.39 (1.4, 6.5)	3.38 (1.4, 6.5)
Caries of permanent teeth	Incidence	Global	28091.58 (24489.9, 31836.2)	28279.35 (24921.6, 31935.5)	28004.83 (24699.7, 31540.3)	29059.00 (25600.9, 32750.2)	29253.43 (25846.5, 32843.4)	29512.68 (26129.2, 33218.0)	29777.03 (26310.2, 33490.9)
		MENA	31592.38 (27499.4, 35933.2)	31636.72 (27408.2, 36060.3)	31394.24 (27534.1, 35525.4)	31405.35 (27464.5, 35494.2)	31748.42 (27634.6, 35968.3)	32008.09 (27963.3, 36123.4)	31920.12 (27848.9, 36314.8)
		Iran	34459.88 (30709.0, 38071.6)	34370.38 (30718.4, 38001.7)	34294.99 (30732.6, 37735.9)	33016.17 (29413.6, 36767.4)	33067.49 (29551.8, 36648.3)	34689.58 (31035.9, 38349.8)	34568.82 (30959.8, 38333.7)
	Prevalence	Global	28331.49 (24315.3, 33176.9)	28207.41 (24524.6, 32731.1)	28161.56 (24524.6, 32667.7)	29076.45 (25525.7, 33546.4)	28562.34 (25028.4, 32987.4)	28424.35 (24801.9, 32916.4)	27543.34 (23976.0, 32018.0)
		MENA	35477.31 (30514.8, 40783.0)	34893.84 (30161.2, 39892.2)	35419.88 (30631.8, 40544.2)	35182.29 (30527.5, 40277.1)	34113.00 (29506.0, 39013.2)	33650.22 (29141.4, 38573.0)	33924.85 (29422.7, 39162.9)
		Iran	32059.87 (28112.7, 36505.7)	31920.79 (28015.9, 36170.3)	32590.88 (28567.9, 36945.9)	33818.34 (29647.4, 38385.3)	33436.66 (29318.7, 37883.2)	31177.03 (27364.9, 35393.2)	31184.50 (27490.5, 35341.8)
	DALY	Global	27.78 (12.2, 52.9)	27.67 (12.3, 52.7)	27.63 (12.2, 52.7)	28.54 (12.6, 54.2)	28.05 (12.4, 53.4)	27.91 (12.3, 53.2)	27.02 (12.1, 51.3)
		MENA	34.79 (15.2, 66.6)	34.22 (15.2, 65.0)	34.74 (15.3, 65.9)	34.51 (15.0, 65.3)	33.45 (14.8, 64.0)	32.99 (14.5, 63.0)	33.22 (14.6, 63.7)
		Iran	31.37 (14.1, 59.9)	31.25 (14.1, 59.6)	31.95 (14.4, 61.2)	33.20 (14.9, 63.9)	32.79 (14.8, 63.2)	30.53 (13.7, 58.5)	30.50 (13.6, 58.8)
Oral disorders	Incidence	Global	48780.54 (42279.3, 57068.8)	48694.77 (42280.0, 56914.3)	48299.34 (42001.4, 56392.4)	48848.65 (42536.7, 56500.5)	48603.15 (42444.7, 55997.7)	48905.75 (42771.5, 56197.4)	48932.66 (43117.3, 55386.5)
		MENA	52335.49 (45181.5, 61374.0)	52243.80 (45278.5, 61217.1)	51682.58 (45155.7, 60333.4)	50309.88 (44385.5, 56921.7)	49164.88 (43120.4, 55383.5)	49569.35 (43868.4, 55618.8)	49544.48 (43621.8, 55914.7)
		Iran	56738.85 (49557.9, 65438.4)	56252.24 (49122.0, 65319.6)	56122.29 (49162.5, 64889.3)	54875.67 (48189.2, 63604.1)	54801.05 (48196.0, 63228.0)	56462.41 (49572.6, 64932.9)	56296.88 (49618.7, 64522.1)
	Prevalence	Global	47044.24 (42842.5, 51317.7)	46539.16 (42549.7, 50590.1)	46319.80 (42468.5, 50211.8)	46999.48 (43277.9, 50875.5)	45972.47 (42432.8, 49788.8)	46529.92 (42967.5, 50431.0)	45908.54 (42261.6, 49783.0)
		MENA	51810.12 (47365.7, 56782.6)	51433.27 (47204.6, 56018.5)	52213.93 (47677.7, 56881.7)	52208.25 (47982.7, 56709.4)	51201.81 (47021.0, 55697.6)	50834.44 (46648.3, 55320.8)	51038.66 (46772.7, 55705.2)
		Iran	50802.02 (46980.7, 54804.9)	50511.76 (46747.3, 54483.8)	50584.37 (46806.4, 54568.0)	52204.29 (48280.5, 56245.1)	51633.29 (47710.4, 55723.1)	50185.76 (46443.4, 53996.1)	50184.34 (46512.8, 54078.9)
	DALY	Global	286.73 (170.5, 433.0)	278.31 (164.9, 422.9)	273.32 (162.4, 417.8)	278.28 (165.7, 423.6)	274.79 (164.9, 412.9)	273.21 (161.5, 416.0)	275.91 (164.1, 416.7)
		MENA	330.28 (198.0, 498.0)	329.71 (196.1, 502.2)	336.52 (196.1, 509.1)	342.89 (206.5, 512.4)	329.05 (197.2, 495.1)	327.90 (195.0, 494.3)	327.56 (193.5, 494.3)
		Iran	314.98 (186.5, 476.3)	312.62 (184.3, 473.5)	315.64 (184.8, 481.5)	310.35 (183.3, 471.1)	279.77 (165.5, 426.3)	317.99 (192.9, 471.2)	318.38 (192.9, 471.0)

MENA, Middle East and North Africa (MENA).

**Figure 1:**
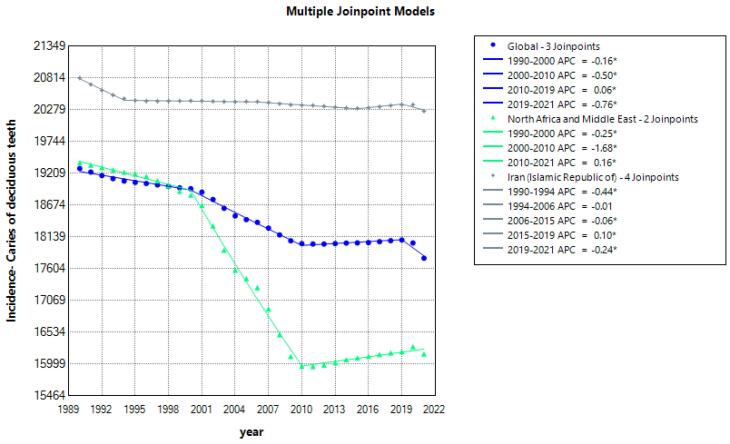
Multiple Joinpoint Regression Model of the Incidence Rate of Deciduous Teeth Caries in Three Locations between 1989 and 2021 Based on the Global Burden of Disease Database

Table 2 (Figure 1) presents findings from the joinpoint regression model analyzing the incidence trends of caries in deciduous teeth. This analysis reveals notable changes in incidence rates over time, highlighting periods of significant decline and stability amidst fluctuating trends. Understanding these patterns is crucial for identifying periods of effective intervention and potential factors influencing dental health outcomes globally. Figure S1 illustrates the heat map for incidence trends of caries in deciduous teeth.

**Table 2:** The j Joinpoint Regression Model Output for the Incidence Rate Trend Analysis of Deciduous Teeth Caries in Three Locations between 1990 and 2021 Based on the Global Burden of Disease Database

**Segments**	**Global**		**North Africa and Middle East**		**Iran**	
	**Time interval**	**APC (95% CI)**	**Time interval**	**APC (95% CI)**	**Time interval**	**APC (95% CI)**
Trend 1	1990-2000	-0.163^*^ (-0.20, -0.12)	1990-2000	-0.252^*^ (-0.32, -0.18)	1990-1994	-0.440^*^ (-0.47, -0.41)
Trend 2	2000-2010	-0.504^*^ (-0.56, -0.46)	2000-2010	-1.684^*^ (-1.74, -1.63)	1994-2006	-0.012 (-0.02, 0.00)
Trend 3	2010-2019	0.060^*^ (0.02, 0.12)	2010-2021	0.160^*^ (0.11, 0.21)	2006-2015	-0.062^*^ (-0.10, -0.05)
Trend 4	2019-2021	-0.760^*^ (-1.00, -0.42)	-	-	2015-2019	0.097^*^ (0.05, 0.16)
Trend 5	-	-	-	-	2019-2021	-0.243^*^ (-0.32, -0.14)
AAPC	1990-2021	-0.250^*^ (-0.26, -0.23)	1990-2021	-0.571^*^ (-0.59, -0.55)	1990-2021	-0.083^*^ (-0.09, -0.08)

^*^Significant at 0.05 level.

**Figure 2:**
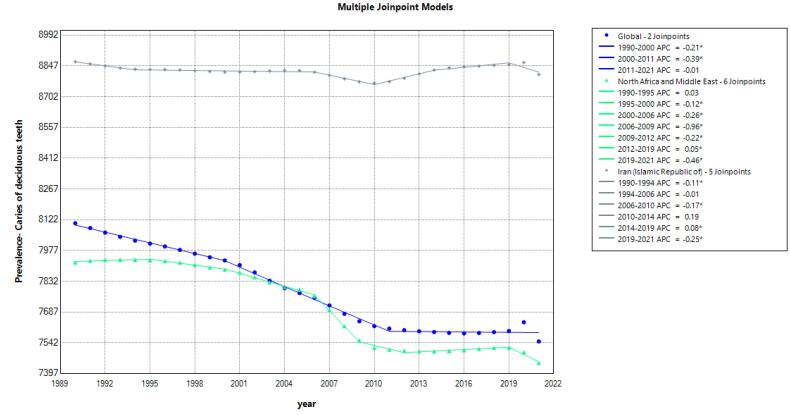
Multiple Joinpoint Regression Model of the Prevalence Rate of Deciduous Teeth Caries in Three Locations between 1989 and 2021 Based on the Global Burden of Disease Database

 Similarly, Figure 2 (Supplementary file 1, Table S1) provides insights into the prevalence trends of deciduous teeth caries, showcasing a gradual decline globally from 1990 to 2011, with subsequent stabilization up to 2021. Noteworthy regional variations are observed, particularly in MENA, where a substantial reduction in caries prevalence occurred from 2006 to 2009. This region also experienced intermittent increases in prevalence during earlier and later segments, underscoring the complex nature of disease dynamics influenced by socioeconomic factors, healthcare access, and oral health practices.

**Figure 3:**
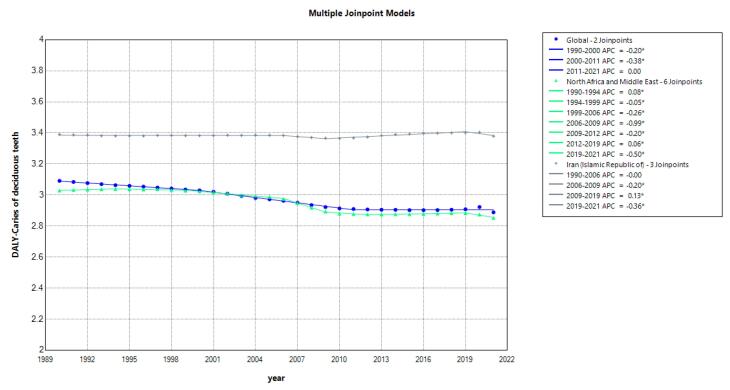
Multiple Joinpoint Regression Model of the DALY Rate of Deciduous Teeth Caries in Three Locations between 1989 and 2021 Based on the Global Burden of Disease Database

Figure 3 (Table S2) illustrates the analysis of DALY trends associated with caries in deciduous teeth. DALYs provide a measure of the overall burden of disease, combining years of life lost due to premature mortality and years lived with disability. The consistent downward trend in DALYs in MENA, despite a slight increase from 1990 to 1994, reflects improvements in healthcare infrastructure and oral health interventions. In contrast, Iran exhibits fluctuating DALY trends, indicating variable impacts on quality of life and healthcare resources allocated to managing dental caries in deciduous teeth.

 The incidence trends of caries in permanent teeth, analyzed in Figure S2 (Table S3), depict a global increase over the study period, punctuated by declines from 1995 to 2000 and 2019 to 2021. These fluctuations suggest varying levels of preventive measures and treatment efficacy across different time segments and geographical regions. MENA shows a predominant upward trend in incidence rates, interspersed with minor declines, highlighting ongoing challenges in addressing oral health disparities and access to dental care. There was a statistically significant difference between the three sites in terms of incidence (*P* < 0.001).

Figure S3 (Table S4) details the prevalence trends of caries in permanent teeth, indicating a general decline globally, except for a notable increase during the early 2000s. This increase underscores potential shifts in dietary habits, oral hygiene practices, and preventive strategies that may influence disease prevalence over time. In MENA, a region with consistently high prevalence rates, fluctuations are observed, including a slight rise from 1995 to 2000, reflecting broader health system challenges and socioeconomic influences on oral health outcomes. There were no statistically significant differences between the three sites in terms of prevalence (*P* = 0.0.078)

 DALY rates associated with caries in permanent teeth, analyzed in Figure S4 (Table S5), reveal a global increase in the early 2000s followed by a decline in subsequent years. This pattern reflects advancements in dental care and public health interventions aimed at reducing the burden of oral diseases. MENA exhibits a fluctuating pattern in DALY rates, indicative of varying disease management strategies and healthcare access across the region. Iran’s DALY trends show a pronounced increase from 1996 to 2004, potentially influenced by changes in disease reporting practices and healthcare infrastructure development. The three sites did not have a statistically significant difference in terms of DALY (*P* = 0.407). While the study focuses on MENA, Iran, and global trends, we noted that MENA’s DALYs for permanent teeth caries (33.22/100 000 in 2021) exceed South Asia’s (28.15/100 000), likely due to weaker preventive infrastructure.

Figure S5 (Table S6) presents the incidence rate trends of oral disorders from 1990 to 2021, highlighting global fluctuations in disease incidence. These variations may be influenced by changes in risk factors such as diet, oral hygiene practices, and access to healthcare services. In MENA, persistent declines in incidence rates are noted, except for the period from 2010 to 2021, suggesting ongoing challenges in maintaining consistent oral health outcomes despite overall improvements in healthcare delivery.

 The prevalence rate trends of oral disorders, detailed in Figure S6 (Table S7), demonstrate global fluctuations with periods of increase followed by declines around 2005 in certain regions. These trends reflect the complex interplay of biological, social, and environmental factors contributing to the prevalence of oral disorders worldwide. Regional variations in prevalence rates underscore the need for targeted public health interventions tailored to local contexts and epidemiological patterns.

Figure S7 (Table S8) describes the DALY rate trends for oral disorders from 1990 to 2021, highlighting regional disparities in disease burden. MENA consistently reports higher DALY rates compared to other regions, indicative of ongoing challenges in disease prevention and healthcare access. Global DALY rates show a slight decrease over time, reflecting improvements in disease management and health promotion efforts globally. The fluctuating trends observed in Iran from 2004 to 2015 may be attributed to variations in healthcare reporting practices and data availability, emphasizing the importance of robust epidemiological surveillance systems.

 While age-standardized rates were used, future analyses should stratify by age and gender. For example, GBD data indicate that women in MENA report 20% higher untreated caries rates than men, possibly due to caregiving-related dietary habits.

## Discussion

 This comprehensive study investigates the epidemiological trends of dental caries and oral disorders over a 31-year period, utilizing data from the GBD studies. The findings reveal significant regional disparities in the incidence, prevalence, and DALY associated with these conditions, highlighting the persistent challenges in achieving equitable oral health outcomes globally.

 Globally, the burden of dental caries and oral disorders remains substantial despite advancements in dental care and preventive measures. The data indicate a general decline in the prevalence of dental caries in deciduous teeth, likely due to improved public health initiatives and increased awareness of oral hygiene practices. However, the prevalence of dental caries in permanent teeth shows more variability, with notable fluctuations over the study period. This aligns with the findings of Kassebaum et al.^15^ indicating that the global burden of dental caries continues to be influenced by factors such as dietary habits, socioeconomic conditions, and access to dental care.

 The global decline in dental caries in deciduous teeth suggests that public health interventions targeting young children have been effective.^13,16^ Programs promoting fluoride use, dental sealants, and educational campaigns about oral hygiene have likely contributed to this trend. However, the rise in dental caries in permanent teeth during certain periods indicates that these interventions may not be reaching all age groups equally or that other factors, such as changes in diet and oral hygiene practices, are counteracting these efforts. This calls for a reassessment of current strategies and the development of more comprehensive approaches that address all stages of life.

 Governments should: (1) allocate 5% of national health budgets to oral health; (2) mandate sugar labeling and taxation; (3) train primary care workers in basic dental screening; (4) establish mobile clinics in conflict zones.

 The study highlights pronounced regional disparities, particularly in MENA and Iran. These regions exhibit higher incidence and prevalence rates of dental caries and oral disorders compared to the global average. The joinpoint regression analysis reveals significant changes in trends over time, with periods of both decline and increase in disease burden. These fluctuations are likely influenced by varying socioeconomic conditions, healthcare infrastructure, and public health policies across these regions.

 In MENA, the incidence and prevalence of dental caries in deciduous teeth have shown significant decline, particularly from 2006 to 2009. However, the overall burden remains high, reflecting ongoing challenges in oral health management. Factors such as limited access to dental care, inadequate public health infrastructure, and social and economic disparities contribute to these disparities.^17^ The persistent inequalities underscore the critical need for targeted interventions such as educational procedures and improved access to preventive dental care and treatment services. Educational messages should consider social and economic contexts and respect cultural differences to be effective.^18^ The prevalence of caries in permanent teeth in MENA (34 924/100 000) is 22% higher than the global average (27 543/100 000), contrasting with declines in Europe. This gap reflects Europe’s robust sugar taxation policies, which reduce consumption by 18%, versus MENA’s limited regulation.

 Healthcare systems in MENA often face challenges such as inadequate funding, insufficient healthcare workforce, and limited availability of dental materials and equipment.^19^ These issues are compounded by high population growth rates and, in some cases, political instability. Improving oral health in these regions requires addressing these systemic issues, alongside implementing targeted public health interventions. Strategies could include increasing investment in healthcare infrastructure, training more dental professionals, and ensuring the availability of essential dental care supplies.

 Iran exhibits a unique trend, with fluctuating DALY rates associated with dental caries and oral disorders. These variations may be linked to changes in healthcare reporting practices, disease management strategies, and broader socioeconomic context.^20^ The pronounced increase in DALY rates from 1996 to 2004 suggests a period of heightened disease burden, potentially exacerbated by limitations in healthcare resources and infrastructure. Economic sanctions and political changes during this period likely impacted the availability and quality of dental care, leading to increased disease burden.

 The fluctuating trends in Iran also highlight the impact of broader socioeconomic factors on oral health outcomes. Economic downturns, changes in healthcare policy, and shifts in public health priorities can all influence the availability and quality of dental care services.^21,22^ Addressing these issues requires a multifaceted approach that includes economic and political reforms, alongside targeted health interventions.

 Sociopolitical factors, including conflicts and economic instability, significantly influence oral health outcomes.^23^ The study suggests that demographic shifts and reduced sugar intake, influenced by conflicts, have contributed to a decrease in the global burden of oral conditions since 1990. These findings highlight the complex interplay between sociopolitical environments and public health outcomes, emphasizing the need for robust health systems capable of adapting to and mitigating these influences.

 Conflicts and economic instability can disrupt healthcare systems, limit access to care, and increase stress, which reduces focus on preventive health behaviors.^24^ During periods of conflict, healthcare resources are often redirected towards emergency and trauma care, leaving preventive services, including dental care, underfunded and understaffed. Additionally, economic instability can reduce individuals’ ability to afford dental care, leading to increased prevalence of untreated dental conditions.

 In Syria, 60% of dental facilities were destroyed during the civil war (2011–2020), leading to a 50% rise in pediatric extractions. Such disruptions underscore the need for conflict-sensitive oral health strategies, like emergency dental kits for displaced populations.

 Cultural factors significantly influence dental caries by shaping dietary habits, oral hygiene practices, and attitudes toward dental care. In regions like MENA, traditional diets high in sugar and limited awareness of preventive care exacerbate dental caries. Effective public health interventions must be culturally tailored to local customs and socioeconomic conditions. Educational initiatives that respect cultural values are essential for improving oral health practices and access to dental care.^18,25^

 Iran’s DALY fluctuations (e.g. a 15% rise during 2004–2010) coincide with phased implementation of the Health Transformation Plan, which initially prioritized emergency care over preventive dentistry. Post-2014 insurance expansions improved access but also revealed previously unmet demand, temporarily inflating the reported burdens.

## Conclusion

 This study underscores the considerable variation in dental caries and oral disorders among different regions, emphasizing the ongoing public health challenges. The persistent disparities in MENA and Iran highlight an urgent need for targeted interventions and policies aimed at improving oral health equity. Efforts should focus on enhancing access to preventive dental care, strengthening healthcare infrastructure, and promoting public health initiatives tailored to local contexts.^26,27^

 To mitigate the burden of dental caries and oral disorders, it is essential to adopt a multifaceted approach for immediate and long-term actions:

 Immediate actions that include: (1) National salt fluoridation programs by 2025; (2) Tax exemptions for dental imports in sanctioned countries; (3) School-based sealant programs targeting 1 million children annually in MENA.

 And long-term actions including:

1. Enhanced Surveillance and Reporting: Strengthening epidemiological surveillance systems to ensure accurate and comprehensive data collection on oral health outcomes. Improved data collection will enable better tracking of disease trends and the effectiveness of interventions. Training health care providers in standardized reporting practices and investing in health information systems should be considered.^28^
2. Targeted Public Health Interventions: Implementing region-specific preventive measures and treatment programs that address the unique challenges and risk factors in each area. Customized, culturally relevant programs rooted in family and community contexts can effectively tackled the diverse challenges faced by the local population.^29^ For instance, in regions with high sugar consumption, programs promoting dietary changes and sugar alternatives could be effective. Community-based interventions that involve local leaders and stakeholders can also enhance the reach and impact of these programs.
3. Improving Healthcare Access: Expanding access to affordable and quality dental care services, particularly in underserved and vulnerable populations. This includes increasing the number of dental clinics, especially in rural and underserved areas, and providing financial assistance or insurance coverage for dental care.^30^ Mobile dental units and tele-dentistry services can also help reach remote populations.^31^
4. Public Education and Awareness: Promoting awareness of oral hygiene practices and the importance of preventive care through community-based health education programs. Educational campaigns should be culturally tailored and leverage local media and communication channels. School-based programs can also play a crucial role in instilling good oral hygiene habits from a young age.^18^
5. Integration of Oral Health into Primary Care: Prasad *et al*. stated that integrating oral health services into primary healthcare systems is crucial to ensure that dental care is accessible and considered an essential part of overall health.^32^ Training primary care providers in basic dental care and preventive practices can help with early identification and management of dental conditions.
6. Research and Innovation: Encouraging research on new preventive measures, treatment modalities, and healthcare delivery models to improve oral health outcomes. Investment in research can lead to the development of innovative solutions tailored to the specific needs and challenges of different regions.^33^

By addressing these key areas, policymakers and healthcare providers can work towards reducing the global burden of dental caries and oral disorders, ultimately improving oral health outcomes and overall well-being for populations worldwide.^34^ Efforts to reduce oral health disparities require sustained support through strong political will, adequate funding, and international collaboration to ensure optimal oral health for all populations.

 Future research must: (1) evaluate cost-effectiveness of tele-dentistry in MENA; (2) assess gender-specific barriers to care; (3) quantify the impact of water fluoridation in arid regions; (4) explore links between oral health and maternal outcomes.

## Supplementary Files

Supplementary file 1 contains Tables S1-S8 and Figures S1-S7.
