# Supplementary material for: Global Burden of Dental Caries and Oral Disorders: A 31-Year Comparative Analysis of Trends in Iran, North Africa and Middle East
Source: Arch Iran Med. 2025 Jun 1;28(6):324–33. doi: 10.34172/aim.33209 (PMC12305416; doi:10.34172/aim.33209)
Supplement: Supplementary file 1 — contains Tables S1-S8 and Figures S1-S7. [file aim-28-324-s001.pdf]

Arch Iran Med. June 2025;28(6):324-S1
10.34172/aim.33209

**Table S1.** Joinpoint Regression Model Output for the Prevalence Rate Trend Analysis of Deciduous Teeth Caries in Three Locations between 1990 and 2021 based on the Global Burden of Disease Database.

| Segments | Global | | North Africa and Middle East | | Iran | |
|---|---|---|---|---|---|---|
| | Time interval | APC (95% CI) | Time interval | APC (95% CI) | Time interval | APC (95% CI) |
| Trend 1 | 1990-2000 | -0.209* (-0.25, -0.14) | 1990-1995 | 0.026 (-0.01, 0.10) | 1990-1994 | -0.105* (-0.20, -0.05) |
| Trend 2 | 2000-2011 | -0.340* (-0.50, -0.35) | 1995-2000 | -0.116* (-0.17, -0.07) | 1994-2006 | -0.010 (-0.02, 0.03) |
| Trend 3 | 2011-2021 | -0.008 (-0.05, 0.04) | 2000-2006 | -0.260* (-0.30, -0.23) | 2006-2010 | -0.170* (-0.24, -0.02) |
| Trend 4 | - | - | 2006-2009 | -0.960* (-1.00, -0.90) | 2010-2014 | 0.193 (-0.15, 0.25) |
| Trend 5 | - | - | 2009-2012 | -0.225* (-0.27, -0.16) | 2014-2019 | 0.078* (0.01, 0.15) |
| Trend 6 | - | - | 2012-2019 | 0.050* (0.03, 0.08) | 2019-2021 | -0.245* (-0.33, -0.11) |
| Trend 7 | - | - | 2019-2021 | -0.462* (-0.56, -0.32) | - | - |
| AAPC | 1990-2021 | -0.209* (-0.22, -0.19) | 1990-2021 | -0.198* (-0.20, -0.19) | 1990-2021 | -0.017* (-0.02, -0,01) |

*Significant at 0.05 level.

**Table S2.** Joinpoint Regression Model Output for the DALY Rate Trend Analysis of Deciduous Teeth Caries in Three Locations between 1990 and 2021 based on the Global Burden of Disease Database.

| Segments | Global | | North Africa and Middle East | | Iran | |
|---|---|---|---|---|---|---|
| | Time interval | APC (95% CI) | Time interval | APC (95% CI) | Time interval | APC (95% CI) |
| Trend 1 | 1990-2000 | -0.195* (-0.23, -0.13) | 1990-1994 | 0.076* (0.02, 0.17) | 1990-2006 | -0.003 (-0.01, 0.01) |
| Trend 2 | 2000-2011 | -0.382* (-0.47, -0.34) | 1994-1999 | -0.052* (-0.2, -0.01) | 2006-2009 | -0.198* (-0.26, -0.07) |
| Trend 3 | 2011-2021 | 0.000 (-0.04, 0.05) | 1999-2006 | -0.260* (-0.30, -0.23) | 2009-2019 | 0.132* (0.11, 0.18) |
| Trend 4 | - | - | 2006-2009 | -0.986* (-1.03, -0.92) | 2019-2021 | -0.361* (-0.54, -0.16) |
| Trend 5 | - | - | 2009-2012 | -0.198* (-0.24, -0.11) | - | - |
| Trend 6 | - | - | 2012-2019 | 0.057* (0.03, 0.09) | - | - |
| Trend 7 | - | - | 2019-2021 | -0.502* (-0.60, -0.40) | - | - |
| AAPC | 1990-2021 | -0.199* (-0.21, -0.18) | 1990-2021 | -0.191* (-0.20, -0.18) | 1990-2021 | -0.001 (-0.01, 0.00) |

*Significant at 0.05 level.

**Table S3.** Joinpoint Regression Model Output for the Incidence Rate Trend Analysis of Permanent Teeth Caries in Three Locations between 1990 and 2021 based on the Global Burden of Disease Database.

| Segments | Global | | North Africa and Middle East | | Iran | |
|---|---|---|---|---|---|---|
| | Time interval | APC (95% CI) | Time interval | APC (95% CI) | Time interval | APC (95% CI) |
| Trend 1 | 1990-1995 | 0.134* (0.07, 0.21) | 1990-1995 | 0.030* (0.00, 0.05) | 1990-2000 | -0.034* (-0.06, 0.00) |
| Trend 2 | 1995-2000 | -0.246* (-0.32, -0.20) | 1995-2000 | -0.174* (-0.22, -0.14) | 2000-2005 | -0.815* (-0.88, -0.74) |
| Trend 3 | 2000-2005 | 0.794* (0.75, 0.83) | 2000-2005 | 0.003 (-0.5, 0.04) | 2005-2010 | 0.037 (-0.03, 0.11) |
| Trend 4 | 2005-2013 | 0.120* (0.07, 0.15) | 2005-2009 | 0.245* (0.20,0.30) | 2010-2015 | 1.043* (1.00, 1.10) |
| Trend 5 | 2013-2019 | 0.285* (0.24, 0.41) | 2009-2015 | 0.173* (0.10, 0,19) | 2015-2021 | -0.056* (-0.11, 0.00) |
| Trend6 | 2019-2021 | -0.100 (-0.24, 0.09) | 2015-2021 | -0.041* (-0.06, -0.02) | - | - |
| AAPC | 1990-2021 | 0.189* (0.18, 0.20) | 1990-2021 | 0.34* (0.03, 0.04) | 1990-2021 | 0.0194* (0.01, 0.02) |

*Significant at 0.05 level

**Table S4.** Joinpoint Regression Model Output for the Prevalence Rate Trend Analysis of Permanent Teeth Caries in Three Locations between 1990 and 2021 based on the Global Burden of Disease Database.

| Segments | Global | | North Africa and Middle East | | Iran | |
|---|---|---|---|---|---|---|
| | Time interval | APC (95% CI) | Time interval | APC (95% CI) | Time interval | APC (95% CI) |
| Trend 1 | 1990-2000 | -0.071* (-0.09, -0.05) | 1990-1992 | -0.561* (-0.68, -0.36) | 1990-1996 | -0.054 (-0.23, 0.04) |
| Trend 2 | 2000-2005 | -0.710* (0.67, 0.74) | 1992-1995 | -0.212 (-0.28, 0.35) | 1996-2000 | 0.524* (0.03, 0.73) |
| Trend 3 | 2005-2010 | -0.402* (-0.47, -0.35) | 1995-2000 | 0.352 (-0.17, 0.43) | 2000-2005 | 0.785 (-0.22, 0.94) |
| Trend 4 | 2010-2015 | -0.071*(-0.12, 0.00) | 2000-2006 | -0.181* (-0.80, -0.14) | 2005-2010 | -0.245* (-1.46, -0.17) |
| Trend 5 | 2015-2019 | -0.754* (-0.82, -0.70) | 2006-2009 | -0.835* (-0.90, -0.33) | 2010-2015 | -1.464 (-1.54, 0.02) |
| Trend6 | 2019-2021 | -0.088 (-0.25, 0.03) | 2009-2015 | -0.292* (-0.34, -0.17) | 2015-2021 | 0.077 (-0.01, 0.16) |
| Trend 7 | - | - | 2015-2021 | 0.140* (0.09, 0.20) | - | - |
| AAPC | 1990-2021 | -0.146* (-0.09, -0.08) | 1990-2021 | -0.080* (-0.15, -0.14) | 1990-2021 | -0.080* (-0.09, -0.06) |

*Significant at 0.05 level

**Table S5.** Joinpoint Regression Model Output for the DALY Rate Trend Analysis of Permanent Teeth Caries in Three Locations between 1990 and 2021 based on the Global Burden of Disease Database.

| Segments | Global | | North Africa and Middle East | | Iran | |
|---|---|---|---|---|---|---|
| | Time interval | APC (95% CI) | Time interval | APC (95% CI) | Time interval | APC (95% CI) |
| Trend 1 | 1990-2000 | -0.063* (-0.08, -0.04) | 1990-1992 | -0.562* (-0.71, -0.31) | 1990-1996 | -0.045 (-0.14, 0.02) |
| Trend 2 | 2000-2005 | -0.712* (0.70, 0.74) | 1992-1995 | -0.208 (-0.27, 0.37) | 1996-2001 | 0.570* (0.40, 0.66) |
| Trend 3 | 2005-2010 | -0.392* (-0.45, -0.34) | 1995-2000 | 0.340 (-0.14, 0.47) | 2001-2004 | 1.030* (0.84, 1.13) |
| Trend 4 | 2010-2015 | -0.069* (-0.11, 0.00) | 2000-2005 | -0.128* (-0.71, -0.06) | 2004-2008 | -0.050 (-0.14, 0.10) |
| Trend 5 | 2015-2019 | -0.750* (-0.82, -0.70) | 2005-2009 | -0.692* (-0.80, -0.35) | 2008-2011 | -0.560* (-0.68, -0.41) |
| Trend6 | 2019-2021 | -0.149* (-0.31, -0.03) | 2009-2014 | 0.364 (-0.45, 0.10) | 2011-2014 | -2.000* (-2.11, -1.90) |
| Trend 7 | - | - | 2014-2021 | 0.081* (0.02, 0.17) | 2014-2021 | -0.054* (-0.10, 0.00) |
| AAPC | 1990-2021 | -0.087* (-0.09, -0.08) | 1990-2021 | -0.152* (-0.16, -0.14) | 1990-2021 | -0.086*(-0.09, -0.07) |

*Significant at 0.05 level

**Table S6.** Joinpoint Regression Model Output for the Incidence Rate Trend Analysis of Oral Disorders in Three Locations between 1990 and 2021 based on the Global Burden of Disease Database.

| Segments | Global | | North Africa and Middle East | | Iran | |
|---|---|---|---|---|---|---|
| | Time interval | APC (95% CI) | Time interval | APC (95% CI) | Time interval | APC (95% CI) |
| Trend 1 | 1990-1995 | -0.034* (-0.07, 0.07) | 1990-1996 | -0.051 (-0.09, 0.00) | 1990-1994 | -0.199* (-0.33, -0.13) |
| Trend 2 | 1995-2000 | -0.183* (-0.26, -0.14) | 1996-2001 | -0.271* (-0.33, -0.18) | 1994-2000 | -0.028 (-0.06, 0.05) |
| Trend 3 | 2000-2005 | -0.256* (0.21, 0.31) | 2001-2004 | -0.690* (-0.75, -0.57) | 2000-2004 | -0.549* (-0.63, -0.47) |
| Trend 4 | 2005-2010 | -0.126* (-0.18, -0.07) | 2004-2007 | -0.346* (-0.56, -0.28) | 2004-2010 | -0.073* (-0.11, -0.03) |
| Trend 5 | 2010-2016 | 0.137* (0.00, 0.16) | 2007-2010 | -0.561* (-0.62, -0.45) | 2010-2015 | -0.651* (0.60, 0.70) |
| Trend6 | 2016-2019 | 0.271* (0.19, 0.33) | 2010-2015 | -0.207* (0.15, 0.27) | 2015-2019 | 0.036* (0.00, 0.45) |
| Trend 7 | 2019-2021 | -0.383* (-0.48, -0.25) | 2015-2021 | 0.013 (-0.04, 0,05) | 2019-2021 | 0.239 (-0.36, -0.08) |
| AAPC | 1990-2021 | 0.014* (0.00, 0.02) | 1990-2021 | -0.172* (-0.18, -0.16) | 1990-2021 | -0.022* (-0.03, -0.01) |

*Significant at 0.05 level

**Table S7.** Joinpoint Regression Model Output for the Prevalence Rate Trend Analysis of Oral Disorders in Three Locations between 1990 and 2021 based on the Global Burden of Disease Database.

| Segments | Global | | North Africa and Middle East | | Iran | |
|---|---|---|---|---|---|---|
| | Time interval | APC (95% CI) | Time interval | APC (95% CI) | Time interval | APC (95% CI) |
| Trend 1 | 1990-1994 | -0.237* (-0.36, -0.17) | 1990-1993 | -0.222* (-0.32, -0.17) | 1990-1997 | -0.103* (-0.17, -0.07) |
| Trend 2 | 1994-2000 | -0.110 (-0.14, 0.10) | 1993-1996 | 0.019 (-0.03, 0.06) | 1997-2000 | 0.083 (0.00, 0.55) |
| Trend 3 | 2000-2005 | 0.335* (0.30, 0.40) | 1996-1999 | 0.444* (0.39, 0.48) | 2000-2005 | 0.691* (0.65, 0.74) |
| Trend 4 | 2005-2010 | -0.490* (-0.52, -0.45) | 1999-2005 | 0.029* (0.00, 0.05) | 2005-2011 | -0.275* (-0.31, -0.24) |
| Trend 5 | 2010-2015 | 0.288* (0.25, 0.32) | 2005-2009 | -0.450* (-0.50, -0.41) | 2011-2014 | -0.797* (-0.85, -0.69) |
| Trend6 | 2016-2018 | -0.396* (-0.45, -0.32) | 2009-2014 | -0.197* (-0.23, -0.15) | 2014-2021 | -0.010 (-0.04, 0.02) |
| Trend 7 | 2018-2021 | -0.078 (-0.15, 0.06) | 2014-2021 | 0.056* (0.04, 0.07) | - | - |
| AAPC | 1990-2021 | -0.077* (-0.08, -0.07) | 1990-2021 | -0.048* (-0.05, -0.04) | 1990-2021 | -0.044* (-0.04, -0.03) |

*Significant at 0.05 level

**Table S8.** Joinpoint Regression Model Output for the DALY Rate Trend Analysis of Oral Disorders in Three Locations between 1990 and 2021 based on the Global Burden of Disease Database.

| Segments | Global | | North Africa and Middle East | | Iran | |
|---|---|---|---|---|---|---|
| | Time interval | APC (95% CI) | Time interval | APC (95% CI) | Time interval | APC (95% CI) |
| Trend 1 | 1990-1993 | -0.724* (-0.88, -0.63) | 1990-1995 | -0.046 (-0.13, 0.01) | 1990-2004 | 0.042 (-0.02, 0.11) |
| Trend 2 | 1993-2000 | -0.392* (-0.41, -0.35) | 1995-2005 | 0.419* (0.40, 0.44) | 2004-2010 | -2.054* (-2.22, -1.8) |
| Trend 3 | 2000-2005 | 0.419* (0.38, 0.45) | 2005-2010 | -0.900* (-0.93, -0.86) | 2010-2015 | 2.785* (2.56, 3.01) |
| Trend 4 | 2005-2009 | -0.316* (-0.39, -0.26) | 2010-2021 | -0.029* (-0.04, -0.01) | 2015-2021 | -0.074 (-0.28, 0.11) |
| Trend 5 | 2009-2016 | -0.104* (-0.14, -0.05) | - | - | - | - |
| Trend6 | 2016-2021 | 0.224* (0.17, 0.30) | - | - | - | - |
| AAPC | 1990-2021 | -0.120* (-0.12, -0.11) | 1990-2021 | -0.029* (-0.03, -0.02) | 1990-2021 | 0.046* (0.01, 0.07) |

*Significant at 0.05 level

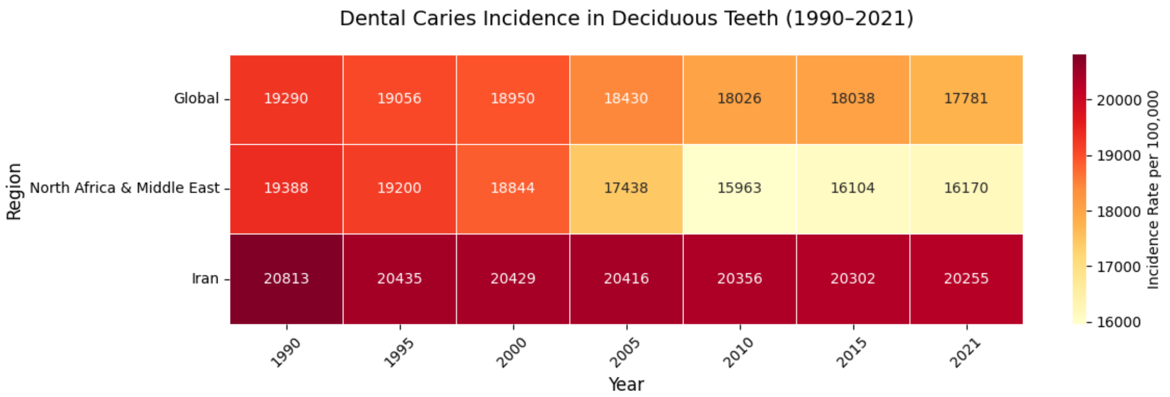


**Figure S1.** Heat Map of Dental Caries Incidence in Deciduous Teeth.

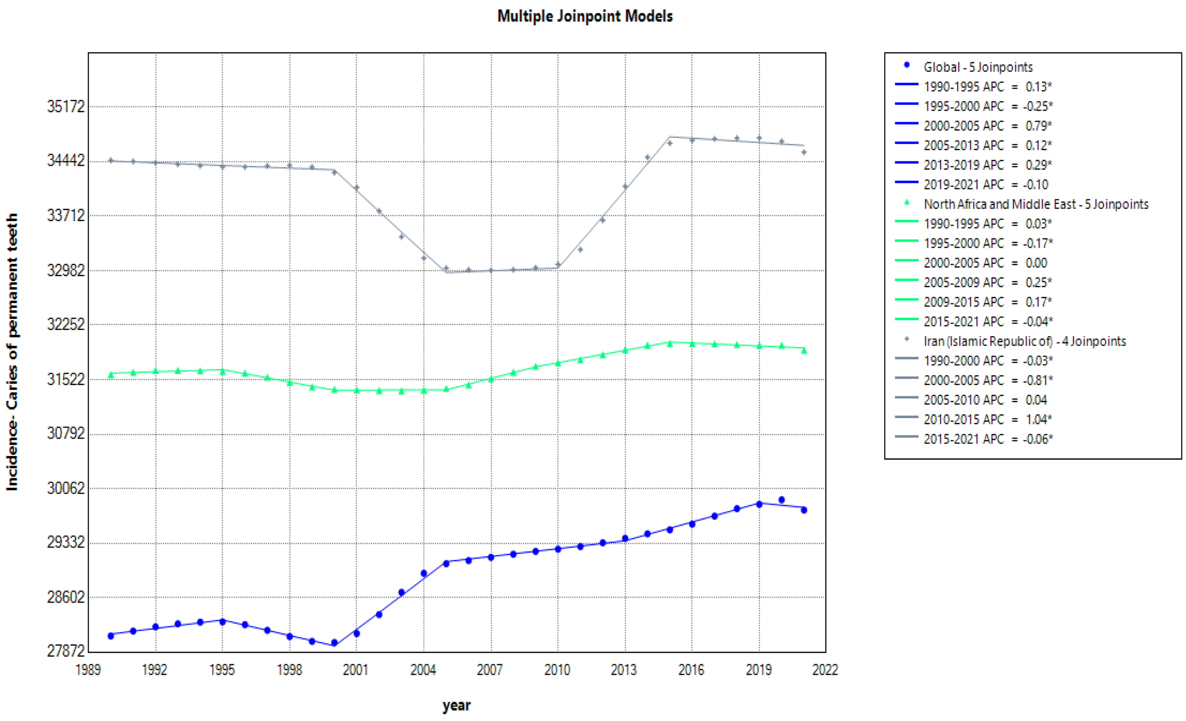


**Figure S2.** Multiple Joinpoint Regression Model of the Incidence Rate of Permanent Teeth Caries in Three Locations between 1990 and 2021 based on the Global Burden of Disease Database.

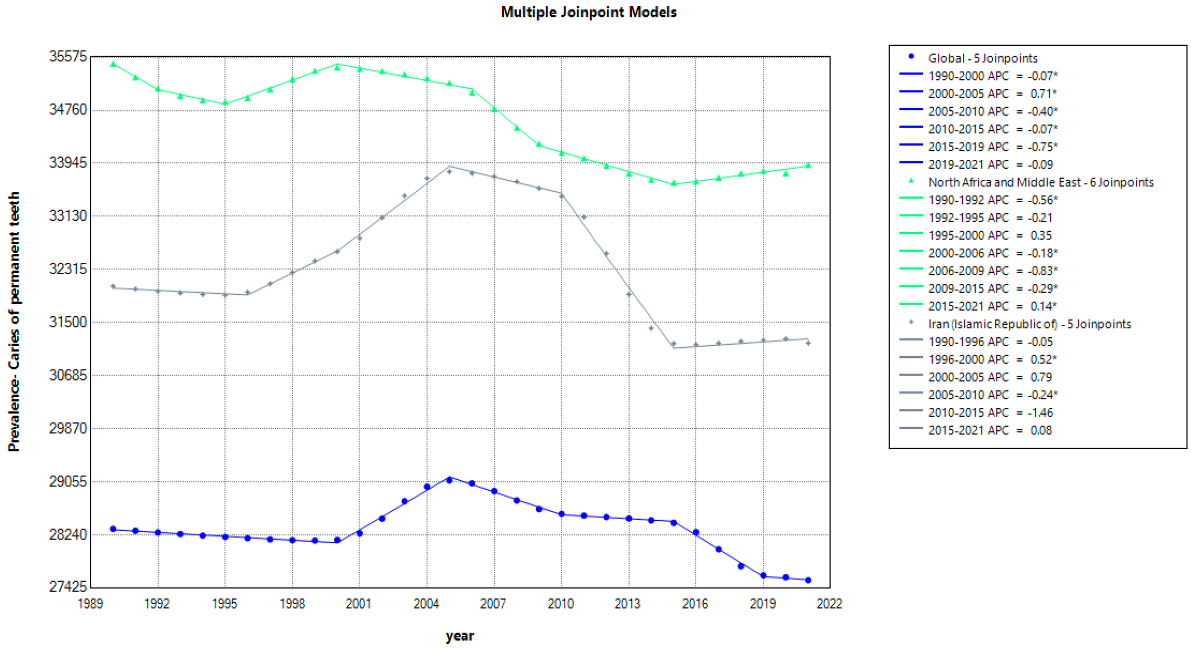


**Figure S3.** Multiple Joinpoint Regression Model of the Prevalence Rate of Permanent Teeth Caries in Three Locations between 1990 and 2021 based on the Global Burden of Disease Database.

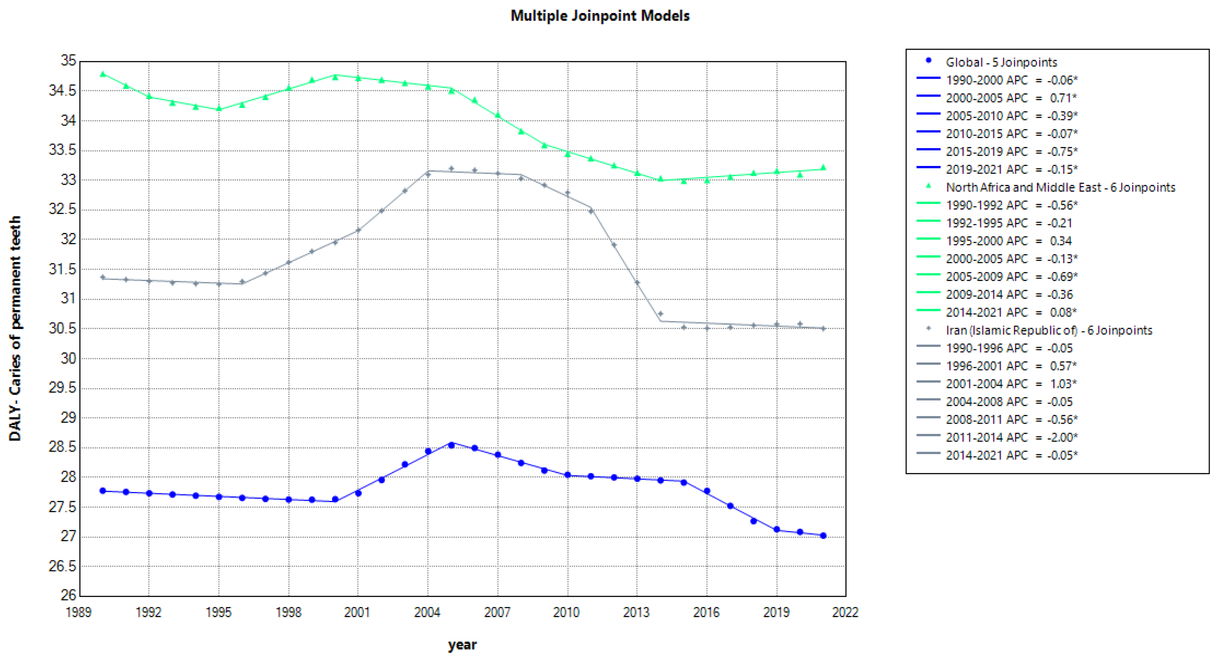


**Figure S4.** Multiple Joinpoint Regression Model of the DALY Rate of Permanent Teeth Caries in Three Locations between 1990 and 2021 based on the Global Burden of Disease Database.

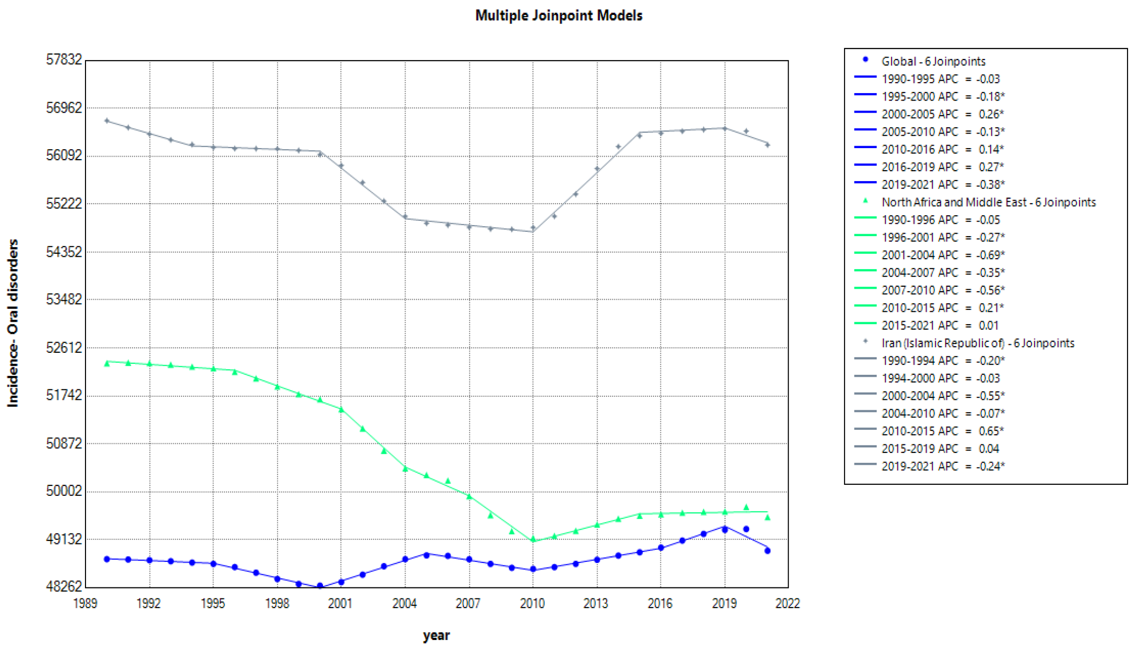


**Figure S5.** Multiple Joinpoint Regression Model of the Incidence Rate of Oral Disorders in Three Locations between 1990 and 2021 based on the Global Burden of Disease Database.

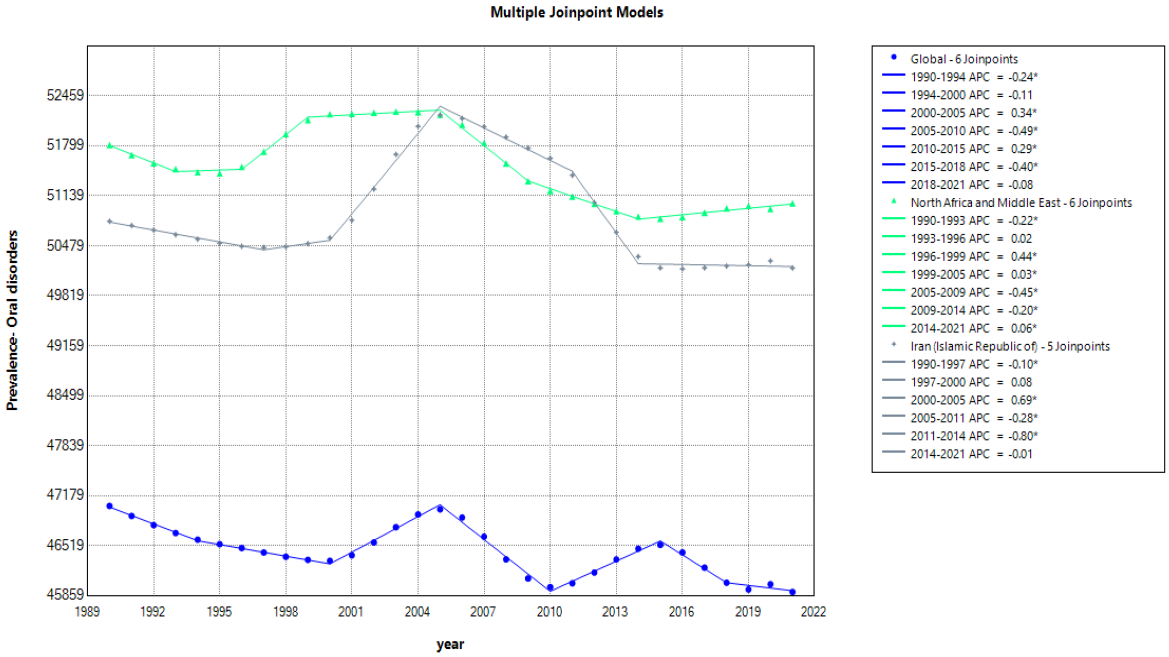


**Figure S6.** Multiple Joinpoint Regression Model of the prevalence Rate of Oral Disorders in Three Locations between 1990 and 2021 based on the Global Burden of Disease Database.

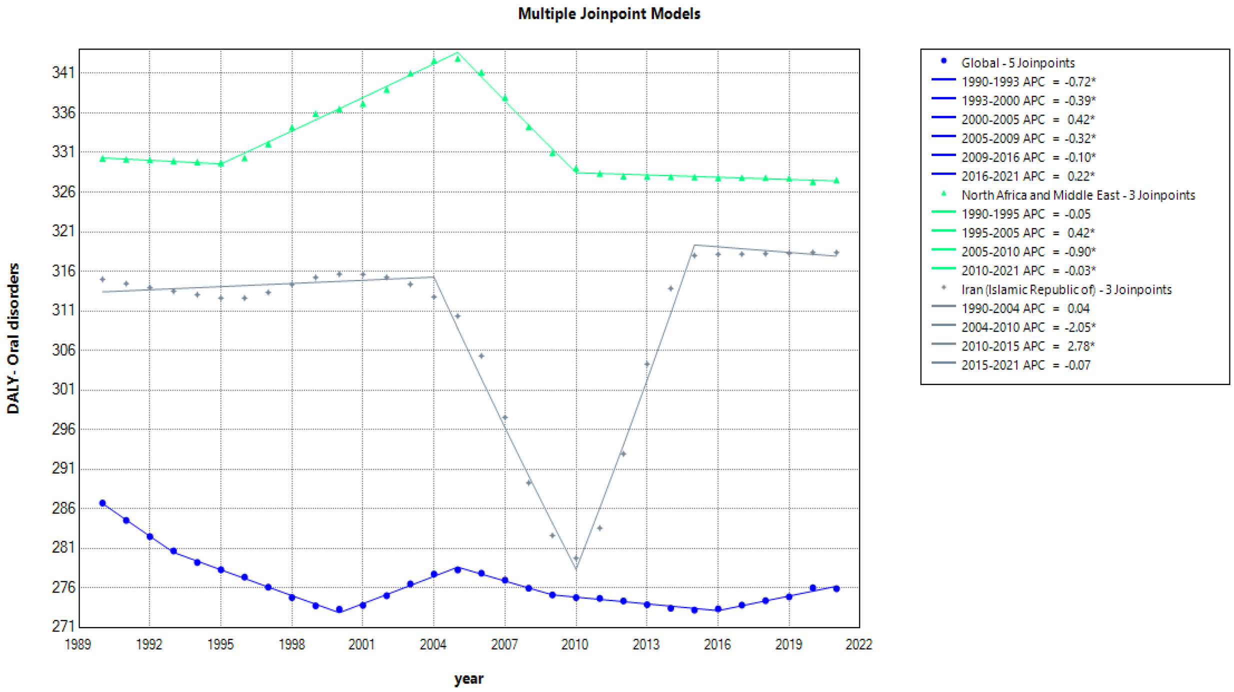


**Figure S7.** Multiple Joinpoint Regression Model Output of the DALY Rate of Oral Deciduous in Three Locations between 1990 and 2021 based on the Global Burden of Disease Database.
